# The Eye of Horus: The Connection Between Art, Medicine, and Mythology in Ancient Egypt

**DOI:** 10.7759/cureus.4731

**Published:** 2019-05-23

**Authors:** Karim ReFaey, Gabriella C Quinones, William Clifton, Shashwat Tripathi, Alfredo Quiñones-Hinojosa

**Affiliations:** 1 Neurosurgery, Mayo Clinic, Jacksonville, USA; 2 Art, University of Miami, Miami, USA; 3 Neurosurgery, University of Texas at Austin, Austin, USA

**Keywords:** ancient egyptian neurosurgery, eye of horus, ancient egypt, neurosurgery, isis and osiris, ancient egyptian mythology, neuroanatomy

## Abstract

Ancient Egyptian civilization is one of the oldest cultures in human history. Ancient Egyptians are well-known for pioneering the fields of art, medicine, and the documentation of discoveries as mythological tales. The Egyptians mastered the integration of anatomy and mythology into artistic symbols and figures. The mythology of Isis, Osiris, and Horus is arguably one of the most recognized mythologies in ancient Egypt. The Eye of Horus was used as a sign of prosperity and protection, derived from the myth of Isis and Osiris. This symbol has an astonishing connection between neuroanatomical structure and function. Artistically, the Eye is comprised of six different parts. From the mythological standpoint, each part of the Eye is considered to be an individual symbol. Additionally, parts of the Eye represent terms in the series 1/2, 1/4, 1/8, 1/16, and 1/32; when this image is superimposed upon a sagittal image of the human brain, it appears that each part corresponds to the anatomic location of a particular human sensorium. In this manuscript, we highlight the possible scientific speculation of the ingenuity of ancient Egyptians’ remarkable insight into human anatomy and physiology.

## Introduction and background

The ancient Egyptian civilization is well-known for many innovations that led to the development of modern systems and utilities that are used daily in the present world [1]. Among these innovations are discoveries in human anatomy and medicine that have led to surgical techniques and instruments still commonly used today. The Egyptians documented many of their findings by combining mythology and mysticism with facts.

We conducted a literature review by searching the database of PubMed, National Geographic Magazine, medical and anatomical history books, and Google Scholar using the search terms: neuroanatomy, Eye of Horus, and the neuroanatomical basis for the Eye of Horus. We could not find any original articles or attempts revealing the neuroanatomical origins of the Eye of Horus.

Ancient Egyptians mastered the integration of anatomical knowledge and mythological stories into artistic symbols and figures. Artistically, the Eye is comprised of six different parts. Mythologically, each part is considered to be an individual symbol. Anatomically, each part corresponds with the center of a particular human sensorium. For many years, the Eye of Horus was considered as a symbol of prosperity and protection by the ancient Egyptians, and its legacy continued into modern Egypt as well [2-3]. However, with a closer look at its artistic design and understanding the epic story behind its creation, the Eye’s current perception as a singular mythologic symbol will be transformed into a powerful example of the ancient Egyptians’ detailed understanding of human anatomy and physiology.

Background and mythology

The Eye of Horus mythology begins with the story of Osiris [4]. This story is the most recognized mythology in ancient Egypt [5]. It illustrates the eternal fight between the virtuous, the sinful, and the punishment [6]. Osiris was the oldest son of the God of the Earth, Geb, and the Goddess of the Sky, Nut, and was known as the God of the Underworld but, more appropriately, as the God of Transition, Resurrection, and Regeneration. Osiris had three siblings: Isis, Set, and Nephthys. Osiris married his sister, Isis, as was the timely Royal custom, and had a son named Horus. The myth started when Set, Osiris’ brother, murdered Osiris to claim the throne, which caused disorder and chaos in ancient Egypt. Set’s brutality did not stop at killing Osiris, and he proceeded to cut Osiris' body into 14 parts that were distributed across ancient Egypt. According to the ancient Egyptian traditions, in order for a royal’s spirit to cross to the underworld, the body needed to be appropriately embalmed and buried in the royal tombs. This proper burial allowed the body to pass through the underworld gates and be judged according to their deeds.

Isis traveled with Horus in search of Osiris’s body parts. Isis also recruited the help of her sister, Nephthys, and Nephthys’ son, Anubis. Anubis was the son of Nephthys and Osiris, and it is said that Nephthys wickedly assumed the shape of Isis to seduce Osiris and conceive Anubis. Isis, Nephthys, Anubis, and Horus were able to find 13 parts of Osiris. The spirit of Osiris was then able to pass to Amenti, the underworld, and rule the dead [7]. When Horus killed Set [8] in the large battle near Edfu, he proclaimed his kingdom, restoring the order to Egypt.

The ancient Egyptians used this legendary fight as a metaphor of the battle between good and evil, order and chaos. Afterward, Horus was idolized by the ancient Egyptians in the form of the Eye of Horus, which was considered as a symbol of prosperity and protection [2-3].

Ancient Egyptians were pioneers in art and medicine. This is exemplified in the artistic measurements of the Eye of Horus. The Eye of Horus was divided into six different parts called the Heqat fractions [9-12], in which each part was considered a symbol itself. The Heqat is among the oldest Egyptian measuring systems in which the numerical values are perceived as a consequential pattern [13]. Gay Robins and Charles Shute discussed this concept in their explanation of the ancient Egyptian mathematical measures of “The Rhind Mathematical Papyrus” [12], which is considered to be the oldest ancient mathematical script. In the Rhind Mathematical Papyrus [13], the Heqat was described as a unit of volume [14], which is used for measurements of goods, such as grain and flour, and it was approximated as 4.8 liters, just over one gallon [15].

The Eye of Horus fragments were organized together to form the whole Eye, similar to the myth, and these fragments were given a series of numerical values with a numerator of one and dominators to the powers of two: 1/2, 1/4, 1/8, 1/16, 1/32, and 1/64 [10-12, 14, 16]. Some historians suggested that each part of the eye represents one of the six senses: smell, sight, thought, hearing, taste, and touch.

**Figure 1:**
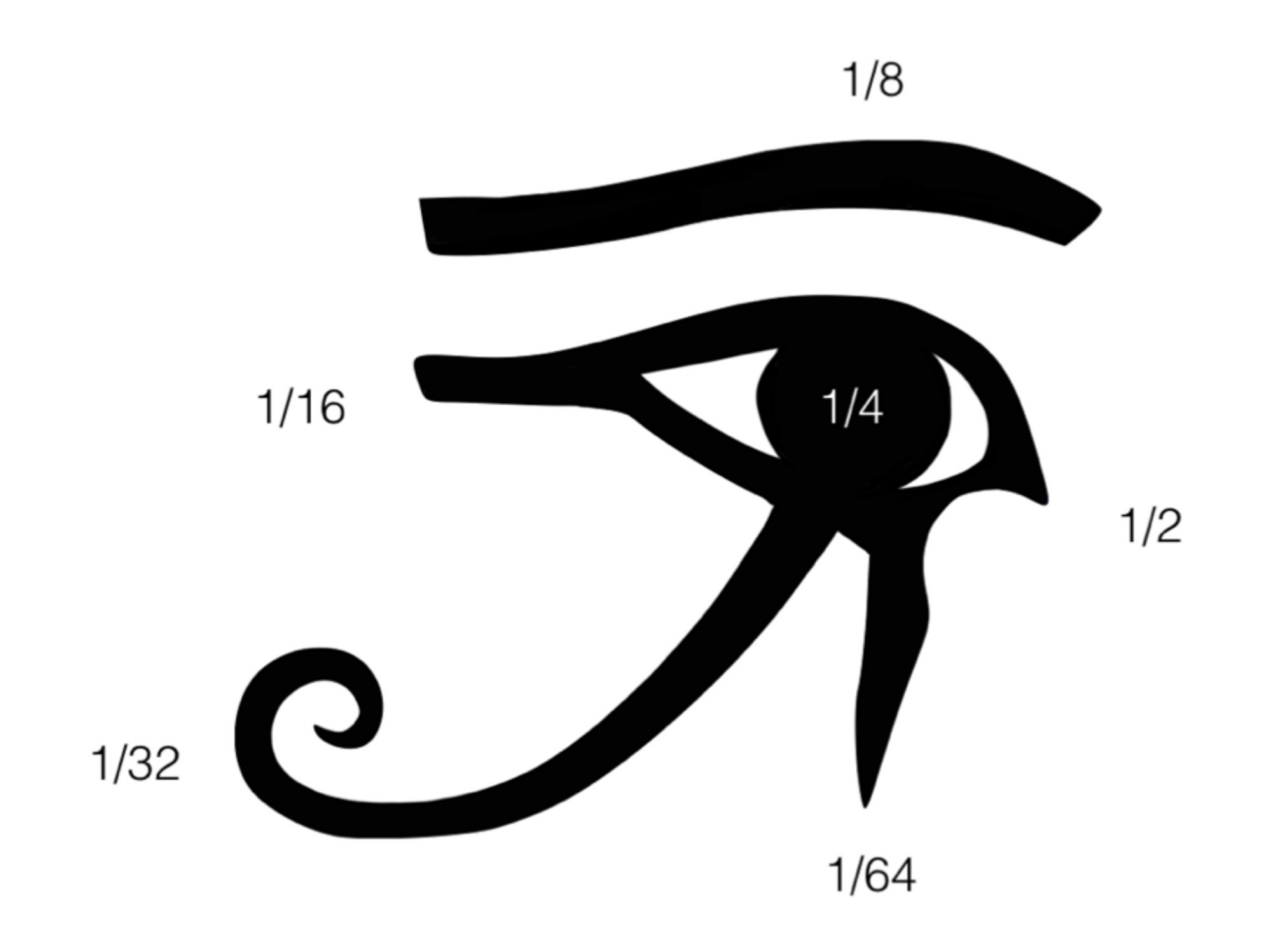
The six mathematical parts of the Eye of Horus The Eye of Horus with its six parts: 1/2, 1/4, 1/8, 1/16, 1/32, and 1/64. The fractions were used to represent the Heqat fractions, the measuring unit that was utilized by the ancient Egyptians for grains and flour, all with powers of two in their denominators and one in their numerator. Each of these fractions corresponds to a different human sense: The 1/2 accounts for the sense of smell, the 1/4 represents sight, the 1/8 represents thought, the 1/16 represents hearing, the 1/32 represents taste, and the 1/64 represents touch.

The 1/2 accounts for the sense of smell, the 1/4 represents sight, the 1/8 represents thought, the 1/16 represents hearing, the 1/32 represents taste, and the 1/64 represents touch (Figure 1) [9-11]. Surprisingly, if we superimposed these suggested parts over the mid-sagittal image of the human brain, each component corresponds to portions of human neuroanatomical features.

## Review

Eye of Horus and its significance to medicine and neuroanatomy

The Eye of Horus has been used for many metaphors over the years, i.e., “Eye of the Mind, Third Eye, Eye of the Truth or Insight, the Eye of God Inside the Human Mind.” The ancient Egyptians, because of their beliefs in the Eye of Horus’ mystic powers, gave all of these names to the Eye of Horus. Herein, we will illustrate the Eye of Horus’ anatomical relevance by observing the series of artwork created by our illustrator (GCQ).

**Figure 2:**
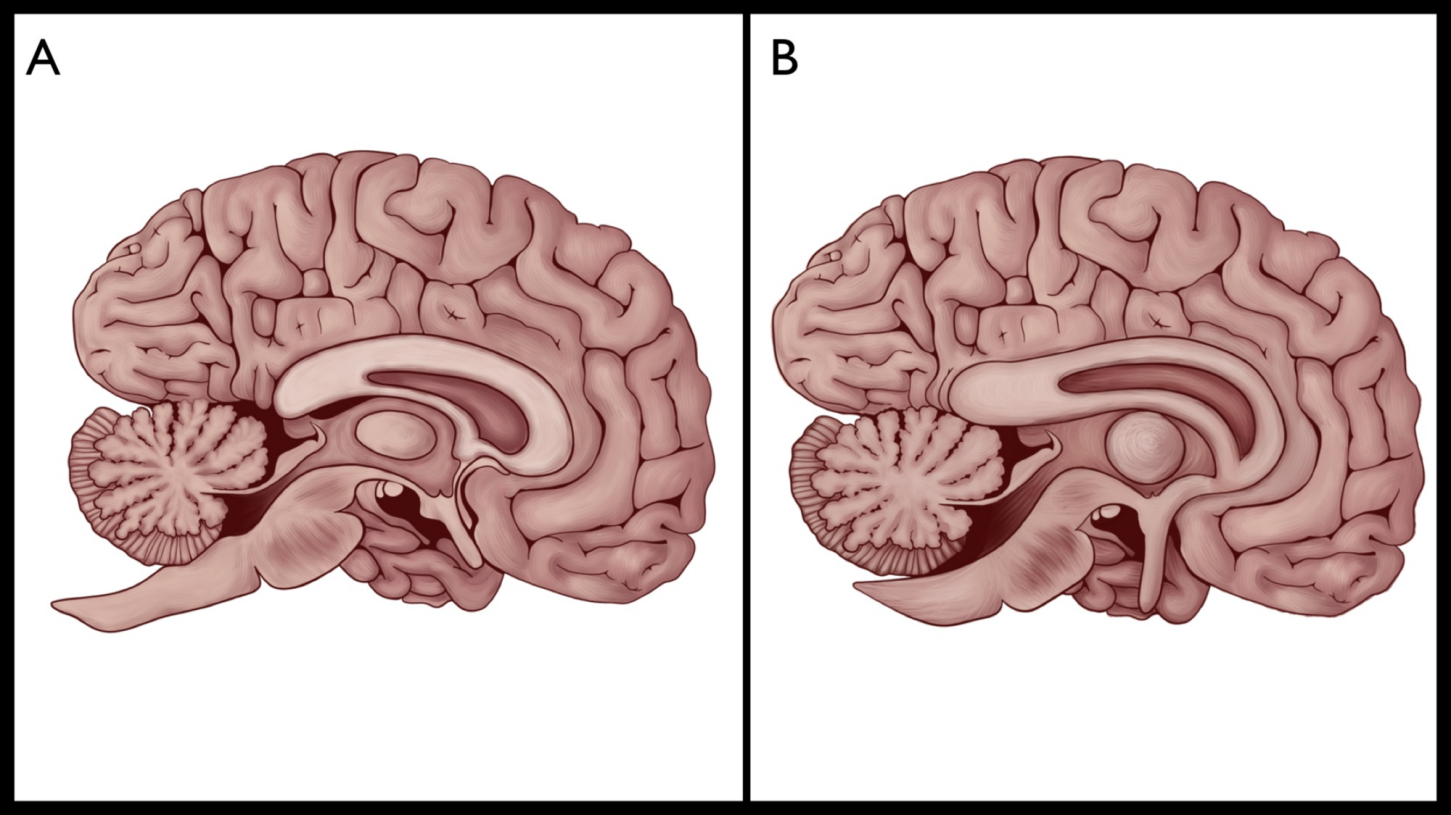
Gross anatomical comparison between the human brain (A), and the human brain described in the ancient ages (B). Figure 2A: Anatomical representation of the mid-sagittal section of the human brain at the level of the corpus callosum and the massa intermedia in the thalamus. Figure 2B: Mid-sagittal section of the human brain with a slight reposition of the direction of the olfactory tract and the orientation of the corpus callosum.

Here, we are trying to highlight one of the most baffling secrets of ancient human history. We are using a modern approach in interpretations of a very old symbol by looking at two images, Figures 2A-2B, and by reviewing the gross neuroanatomy.

**Figure 3:**
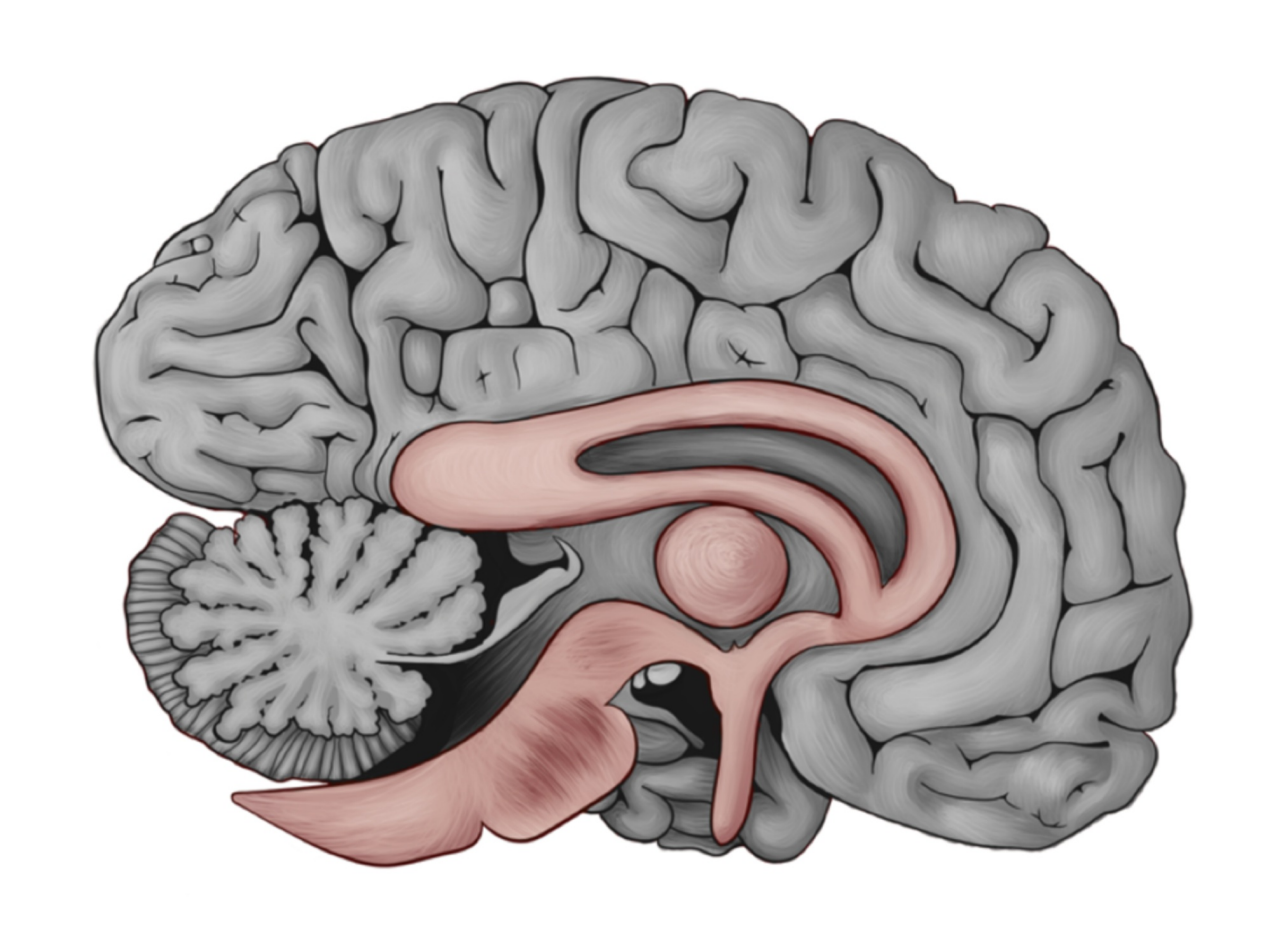
Color differentiated brain between the old and new gross anatomical description of the human brain. The human brain in grey color with the designated area (Figure 2B) highlighted in brown color involving the corpus callosum, metathalamus, olfactory tract, and the brain stem.

We can speculate that there are remarkable similarities between the two images; yet, there are absolute differences in the direction and position of the olfactory tract, as well as the orientation of the corpus callosum. We highlighted these differences in brown and the rest of the brain in grey as shown in Figure 3. 

**Figure 4:**
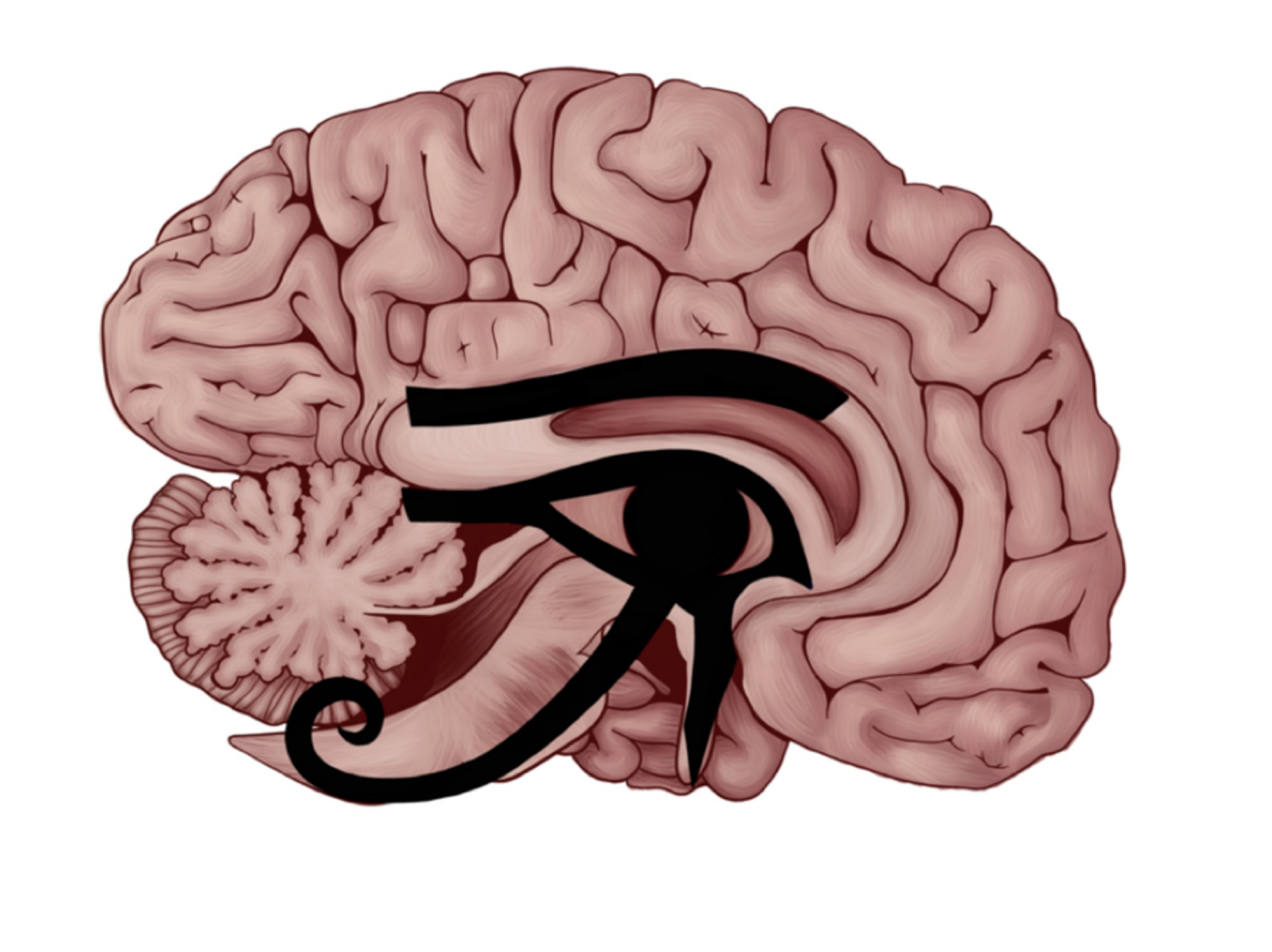
The Eye of Horus fitted in the mid-sagittal section of the human brain. This figure shows the different parts of the Eye of Horus fitting the anatomical structures that carry special brain function depicted by the Heqat fractions.

We thought to add more elaboration by extracting the brown-colored portion in Figure 3 and superimpose the plain eye in Figure 1 on top of it. In Figure 4, we are showing the Eye of Horus entirely fitted on the mid-sagittal section of the human brain. 

Smell: 1/2

**Figure 5:**
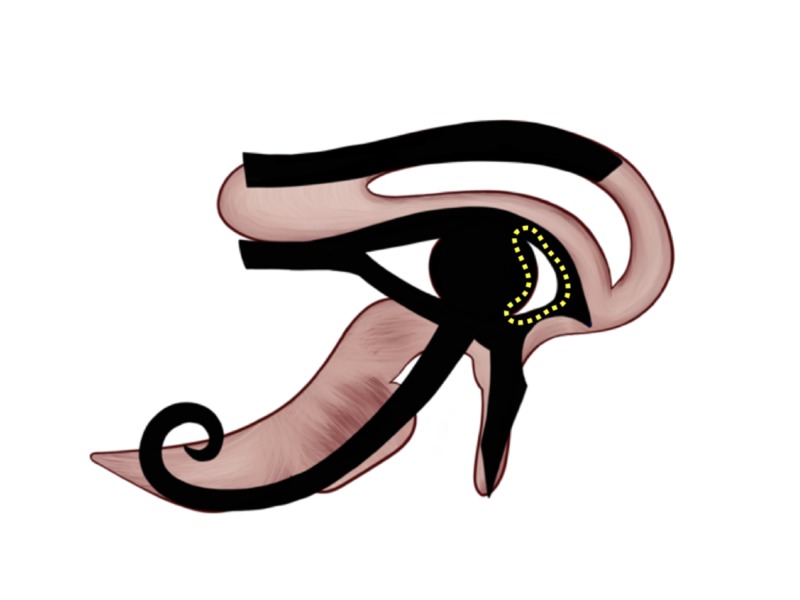
The smell representation of the Eye of Horus Figure 2B was adjusted with the implementation of the Eye of Horus (Figure 1) to highlight the Eye of Horus' triangular-shaped object that resembles the side view of the human nose as a symbol of smell and is located in the location of the olfactory trigone.

To show the significance of the Eye of Horus in human neuroanatomy, we go beyond the visual world and explore the hidden mysteries of the human senses, starting with the sense of smell. On the Eye of Horus, the smell is represented by the triangular shaped object on the right side of the Eye’s pupil, illustrated by the yellow triangle in Figure 5. On a closer look, this triangular-shaped object was designed in a way to resemble the side view of the human nose as a symbol of smell and was given the 1/2 Heqat fraction [10-13]. The 1/2 Heqat fraction [10-13] is also in the identical location and shape of the olfactory trigone.

Vision: 1/4

**Figure 6:**
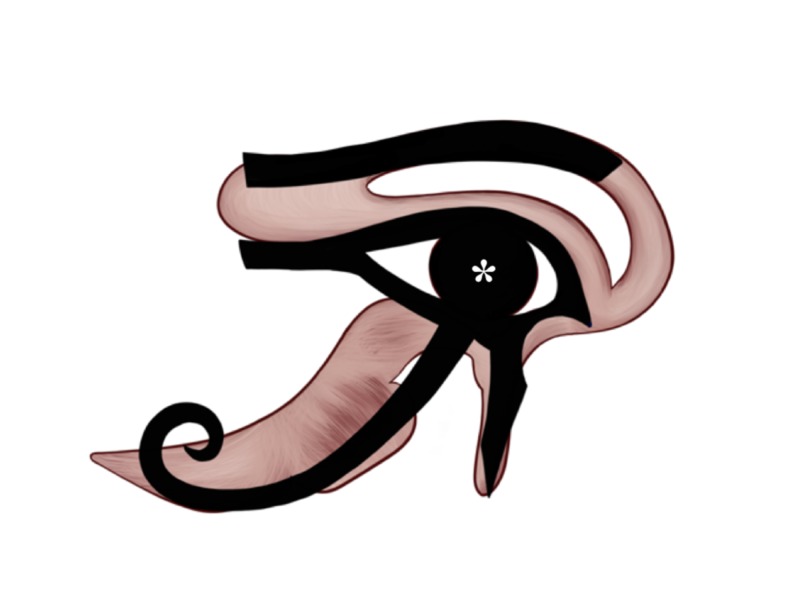
The representation of vision in the Eye of Horus Figure 2B was adjusted with the implementation of the Eye of Horus (Figure 1) to highlight the Eye of Horus' central round-shaped object that resembles the shape and location of the massa intermedia (interthalamic adhesion) and hypothesized as the symbol of vision.

The human perceives vision when the light hits the retina inside the globe, sending neuronal electrical impulses through the optic pathways to the interthalamic adhesion (massa intermedia) where some of the thalamic fibers that carry the vision, along with other sensations, move towards the midline and then curve laterally to the same thalamus. The impulses are sent from the thalamus to the optic radiation tracts and then to the visual cortex in the occipital lobes. On the Eye of Horus, the pupil of the Eye, illustrated by (*) in Figure 6, represents the sight or vision sensation and was given the 1/4 Heqat fraction [10-11, 13]. We hypothesize that the massa intermedia (interthalamic adhesion) was the center of vision; however, we acknowledge that there is no strong evidence to support our hypothesis. The 1/4 Heqat fraction [10-11, 13] is also in the identical location and shape of the massa intermedia (interthalamic adhesion).

Wisdom: 1/8

**Figure 7:**
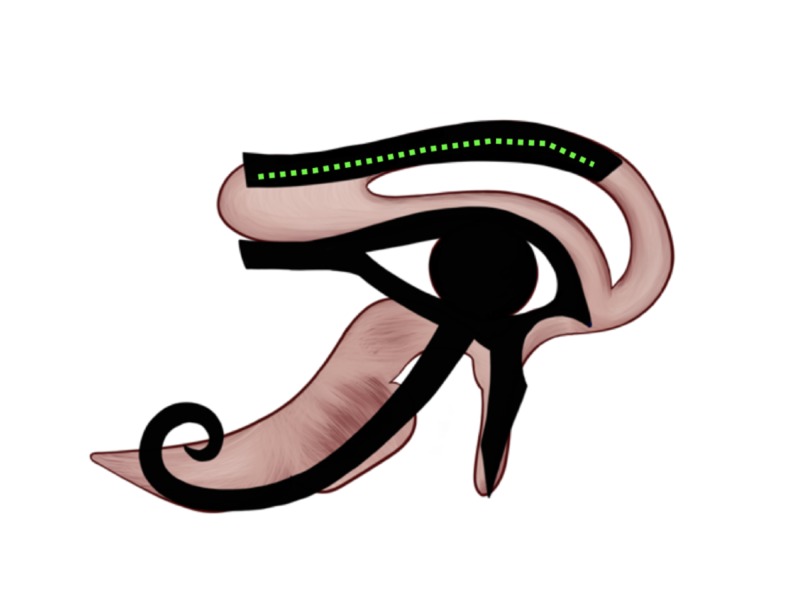
Wisdom/thoughts representation on the Eye of Horus Figure 2B was adjusted with the implementation of the Eye of Horus (Figure 1). The eyebrow-shaped portion of the Eye of Horus resembles the shape and location of the corpus callosum and we hypothesized as the symbol for wisdom or thoughts.

One of the metaphoric names of the Eye of Horus is the Eye of the Mind, which was named after its reputation as the symbol of wisdom or thought. Wisdom is represented by the eyebrow of the Eye and given the 1/8 Heqat fraction [10-11, 13]. The eyebrow is often associated with thinking; for example, we move our eyebrows to express various emotions. From the anatomical perspective, it resembles the corpus callosum. The corpus callosum is the largest collection of the white matter fibers within the brain and facilitates the rapid transmission of neuronal impulses between both hemispheres. On the Eye of Horus, the eyebrow, illustrated by the green dotted line in Figure 7, represents wisdom and was given the 1/8 Heqat fraction [10-11, 13]. The 1/8 Heqat fraction [9-10, 12] exactly resembles the location and shape of the corpus callosum. 

Hearing: 1/16

**Figure 8:**
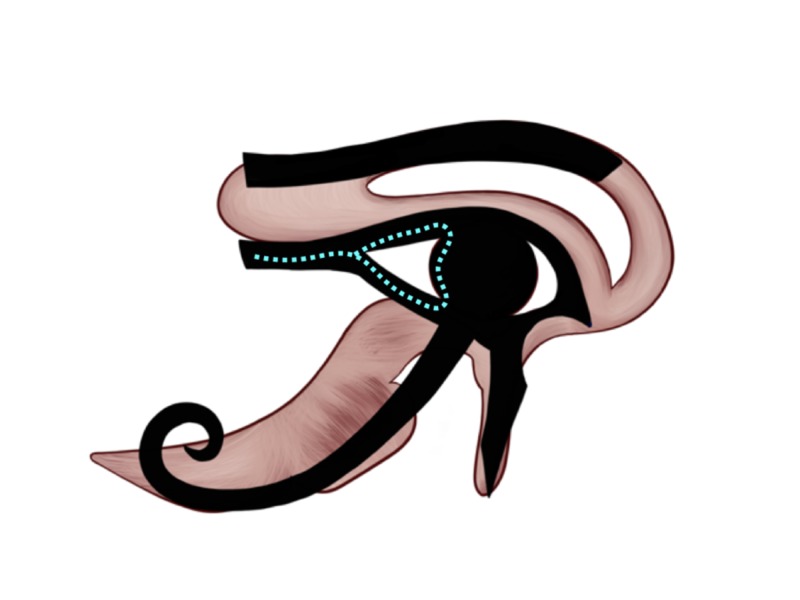
Hearing representation on the Eye of Horus Figure 2B  was adjusted with the implementation of the Eye of Horus (Figure 1) to highlight the triangular-shaped object and the lateral commissure (canthus) on the left side of the Eye of Horus pupil. It resembles the shape and location of the Brodmann areas 41 and 42, which are the center of hearing in humans.

The primary auditory cortex of the temporal lobe is represented by the name Brodmann areas 41 and 42, which located in the anterior transverse temporal lobe (Brodmann area 41) and posterior transverse temporal lobe (Brodmann area 42). On the Eye of Horus, hearing is represented by the triangular-shaped object and the lateral commissure (canthus) on the left side of the Eye’s pupil, illustrated by the dotted cyan triangle and the attached dotted line on Figure 8, and was given the 1/16 Heqat fraction [10-11, 13]. The 1/16 Heqat fraction [10-11, 13] is aligned to the same location and shape of the Brodmann areas 41 and 42. 

Taste: 1/32

**Figure 9:**
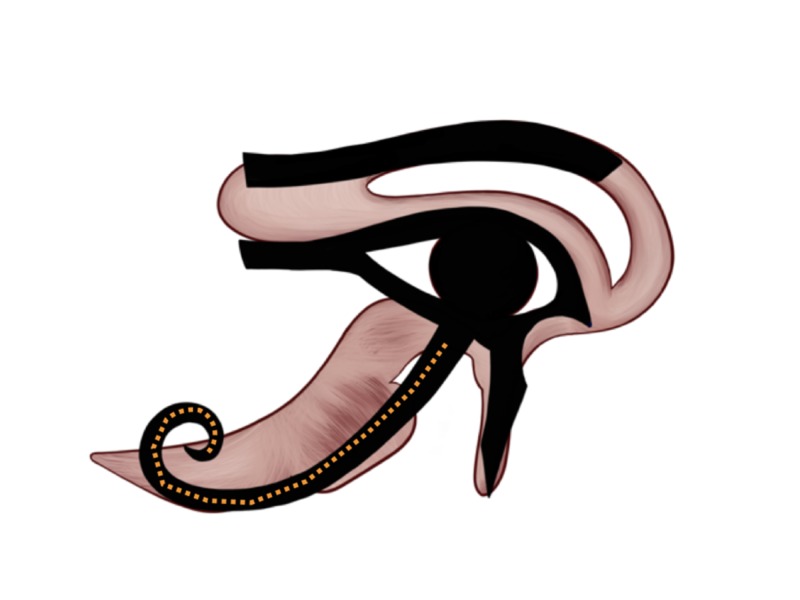
Taste representation on the Eye of Horus Figure 2B  was adjusted with the implementation of the Eye of Horus (Figure 1) to highlight the Eye of Horus' tail-shaped object that resembles the shape and location of the taste pathway in the human brain.

The taste sensation is carried to the thalamus, then to the primary gustatory area of the cerebral cortex for interpretation. On the Eye of Horus, taste is represented by the curved tail, illustrated by the dotted orange curved line on Figure 9, and was given the 1/32 Heqat fraction [10-11, 13]. The 1/32 Heqat fraction [10-11, 13] of the Eye resembles the taste pathway in the human brain. We hypothesize that ancient Egyptians used this fraction as a part of their mystic arts. 

Touch: 1/64

**Figure 10:**
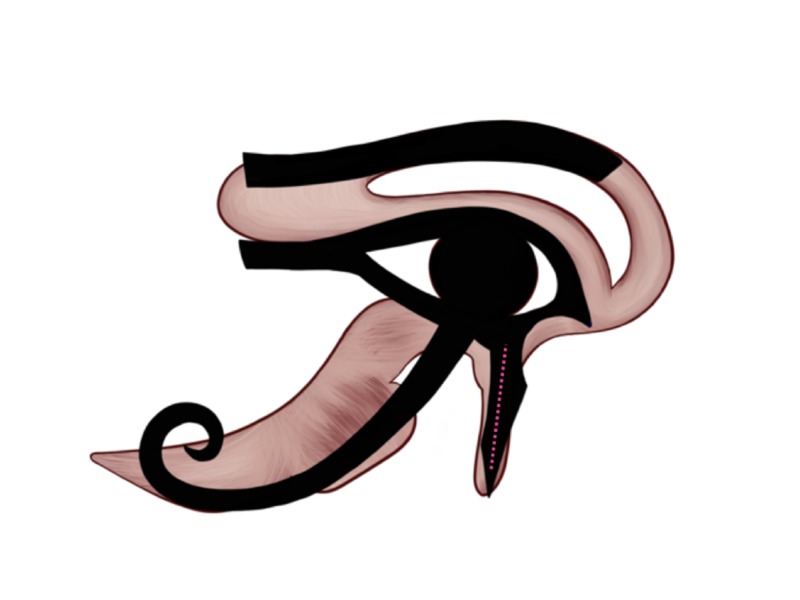
Touch representation on the Eye of Horus Figure 2B was adjusted with the implementation of the Eye of Horus (Figure 1) to highlight the straight object coming down from the right side of the Eye of Horus' pupil. It resembles the shape and location of the somatosensory pathway, which is the carrier of numerous sensations from the body, i.e., light touch, pain, pressure, temperature, joint and muscle position sense (proprioception), to the brain.

Touch sensation is carried by the somatosensory pathway, which carries numerous sensations from the body, i.e., light touch, pain, pressure, temperature, joint and muscle position sense (proprioception). These sensations are divided into three groups, and each group is carried by a different pathway in the spinal cord with a different target in the brain cortex. The first group includes touch, pressure, and vibration perception and allows us to define the shapes and textures of the objects without sight. These senses are carried by the posterior column-medial lemniscus pathway of the spinal cord. The second group includes pain and temperature senses that are carried by the lateral spinothalamic tract. The third group includes proprioception, which allows us to sense the relative position of body parts and the strength needed for movement. On the Eye of Horus, the touch sensation is represented by the straight object coming down from the right side of the Eye, illustrated by the dotted pink line on Figure 10, and was given the 1/64 Heqat fraction [10-11, 13]. The 1/64 Heqat fraction [10-11, 13] of the Eye resembles the somatosensory pathway. 

We acknowledge that a portion of the findings, as well as the hypothesis, are not based on a scientific explanation but rather on a hypothesis based on the history of the ancient Egyptian's mastery in medicine, arts, and mysticism

## Conclusions

Although we recognize the liabilities of overinterpreting a symbolic masterpiece like the Eye of Horus, we propose that the anatomical metaphors in the Eye of Horus are not by coincidence and merit discussion. The ancient Egyptians were leaders in medicine and anatomy. This can be found in documented papyrus, as well as the walls of many temples and tombs. In the creation of Eye of Horus, ancient Egyptians combined their artistic abilities and knowledge of anatomy with their deep belief in mythology. More importantly, we argue that there is a clear influence of their interpretation of human senses on the size and shape of the Eye. This is an amazing feat considering the unavailability of radiographic and computational technology in that era. The significance of our theory of the Eye of Horus is not to be used as an anatomical gold standard but rather to acknowledge and appreciate the genius and foresight of an ancient civilization in decoding the intricate functions of the human central nervous system.
